# Single-Sample Node Entropy for Molecular Transition in Pre-deterioration Stage of Cancer

**DOI:** 10.3389/fbioe.2020.00809

**Published:** 2020-07-14

**Authors:** Chongyin Han, Jiayuan Zhong, Jiaqi Hu, Huisheng Liu, Rui Liu, Fei Ling

**Affiliations:** ^1^School of Biology and Biological Engineering, South China University of Technology, Guangzhou, China; ^2^School of Mathematics, South China University of Technology, Guangzhou, China

**Keywords:** cancer molecular network, single-sample node entropy, critical transition stage, dynamic network biomarker, drug targets

## Abstract

A complex disease, especially cancer, always has pre-deterioration stage during its progression, which is difficult to identify but crucial to drug research and clinical intervention. However, using a few samples to find mechanisms that propel cancer crossing the pre-deterioration stage is still a complex problem. In this study, we successfully developed a novel single-sample model based on node entropy with *a priori* established protein interaction network. Using this model, critical stages were successfully detected in simulation data and four TCGA datasets, indicating its sensitivity and robustness. Besides, compared with the results of the differential analysis, our results showed that most of dynamic network biomarkers identified by node entropy, such as *NKD2* or *DAAM1*, located in upstream in many important cancer-related signaling pathways regulated intergenic signaling within pathways. We also identified some novel prognostic biomarkers such as *PER2*, *TNFSF4*, *MMP13* and *ENO4* using node entropy rather than expression level. More importantly, we found the switch of non-specific pathways related to DNA damage repairing was the main driven force for cancer progression. In conclusion, we have successfully developed a dynamic node entropy model based on single case data to find out tipping point and possible mechanism for cancer progression. These findings may provide new target genes in therapeutic intervention tactics.

## Introduction

Cancer has become the most difficult disease in all medical fields and is one of the leading causes of death in the world (Nordin et al., 2019). Unfortunately, cancer progression can be inconspicuously with non-typical clinical symptoms (Yang et al., 2018; Liu and Shi, 2019). When most patients have pathological symptoms that can be easily diagnosed clinically, they may have already reached the advanced stage (Miller et al., 2019). Missing the best time for clinical intervene makes patients have no choice but to accept some risky therapies. Thus, biomarkers that can improve cancer molecular characteristic is the most important biology signature for both doctors and patients (Shao et al., 2010). Although many targeted drugs have been developed based on this strategy, it must be acknowledged that such result-oriented molecular targets still have failure cases, even the most popular immune checkpoint treatments (Le et al., 2015; Sharma and Allison, 2015). However, such studies based on pairwise differences can only help us to recognize the results that have occurred. Moreover, a recent study suggests that most oncogenetic pathways which support cancer cell survival are already over-active in seeming normal stages (Zielińska and Katanaev, 2019). Normally, intracellular signaling pathways are strictly regulated, yet they have been almost out of control during the cancer progression. Little is known about the reasons for these drastic changes. Since the activities of cells can be characterized by molecules and their interaction within the cell, which is similar to the signal transmission process (Zielińska and Katanaev, 2019). So, we think mathematics and informatics strategy may help us solve these problems.

**FIGURE 1:**
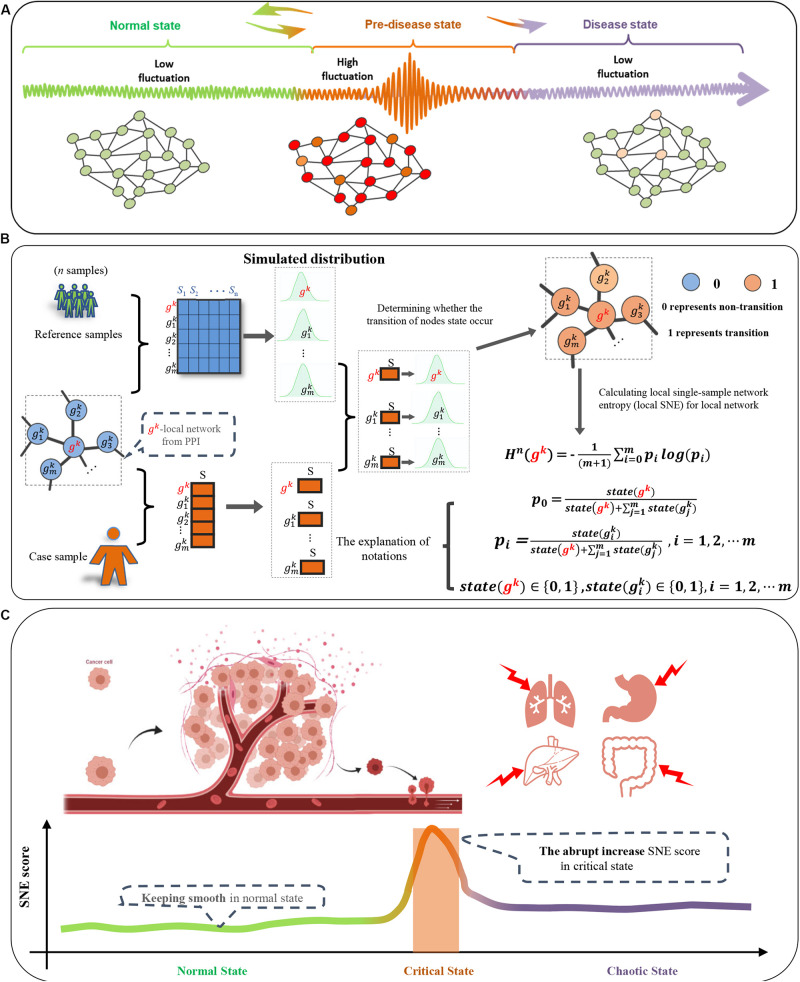
Overall project design together with algorithm details. **(A)** Three-Stage transition of PPI network during disease progression in classic dynamic network biomarker theory; **(B)** one-sample based node entropy algorithm; **(C)** a spike of the SNE curve exists in cancer progression.

Considerable evidence indicates a complex disease is not always smooth process but occasionally abrupt, which means there is a critical transition from a healthy condition to disease state (Chen et al., 2012; Liu et al., 2012). Generally, the disease progression can be roughly divided into three stages (Chen et al., 2012; Liu et al., 2012): a relatively normal state, a critical transition state, and a disease state (Figure 1A). Since cancer is difficult to cure after deterioration, it is very important to identify the critical state before deterioration to enable just-in-time interventions. Identifying such molecular dynamic state for cancer has received considerable attention recently (Liu et al., 2014). A new concept called dynamic network biomarker (DNB) has been successfully employed to detect the critical state and find out molecular activity before disease at the network level (Chen et al., 2012; Liu et al., 2012, 2013). The DNB theory has been applied in the analysis of real biological and clinical data in many research areas, such as the detection of the cancer critical state (Koizumi et al., 2019; Liu et al., 2019, 2020), the cell differentiation (Richard et al., 2016) and the immune checkpoint blockade (Lesterhuis et al., 2017). However, DNB theory requires multiple data samples for each individual to evaluate its three statistical conditions, which significantly restricts its application because multiple samples for every patient is unavailable in many disease studies. In addition, the traditional DNB does not reflect the importance of genes in cellular signaling.

To detect the critical state at network level and to understand the regulatory mechanism of cancer progression, we develop a novel computational method called single-sample node entropy (SNE). The SNE score is constructed to measure the disorder of a local network caused by each individual against the background distribution in terms of gene expression (Figure 1B). This novel computational method has applied to both simulation and real datasets. We applied the method of SNE to the analysis of four different cancer including Esophageal carcinoma (ESCA), Uterine Corpus Endometrial Carcinoma (UCEC), Head and Neck squamous cell carcinoma (HNSC), and Rectum adenocarcinoma (READ) from The Cancer Genome Atlas (TCGA). Our goal is to enables in-depth study of the molecular characteristics of pre-deterioration stage of cancer, as well as provides better clinical treatment targets.

## Materials and Methods

### Algorithm to Identify the Critical Point Using SNE

The SNE method is designed to detect a critical state before a critical transition from the relatively normal state into the advanced state. There exist a group of molecules defined as DNB biomolecules, which satisfy the following the three statistic conditions:

1. *SD*_in_ increases sharply, where *SD*_in_ represents the standard deviation or coefficient of variation for any member in the DNB group;
2. *PCC*_in_ increases sharply, where *PCC*_in_ represents the correlation between any two members in the DNB group;
3. *PCC*_out_ rapidly decreases, where *PCC*_out_ represents the correlation between any one member in the DNB group and any other non-DNB member.

The above three statistic conditions are necessary conditions for phase transition in biological system. According to that, it is obvious that the critical transition of a system is actually quantified by some variables that are strongly fluctuating. The perturbation of local networks of these variables provides the early warning signals of the critical transition. Therefore, the SNE method can identity the critical point by exploring the perturbation triggered by a single case sample to local networks of these variables.

Given a number of reference samples (the normal or control samples which are viewed as the background), the following algorithm is able to identify the critical point with only one single sample from the patient (Figure 1):

[Step 1]. Map the genes to protein-protein interaction (PPI) network defined as a global template network *N*^*G*^. In this work, the PPI network is downloaded from the STRING database, in which all the isolated nodes are discarded. Clearly, each individual’s PPI network is identical as the global template network *N*^*G*^.

[Step 2]. Segment the global template network*N*^*G*^ into different local networks. The global template network *N*^*G*^ is segmented into *Q* local networks, i.e., the local network *N*^*k*^(*k* = 1,2,⋯*Q*). Specifically, for local network *N*^*k*^, it is centered on the gene *g*^*k*^, whose first-order neighbor nodes can be marked as the gene sets $\left\{g_{1}^{k},g_{2}^{k},\ldots ,g_{m}^{k}\right\}$.

[Step 3]. Fit a distribution for each gene of each local network *N*^*k*^(*k* = 1,2,…*Q*) in terms of the expressions from the reference samples. Specifically, for a gene $g_{i}^{k}$ from the local network *N*^*k*^, its Gaussian distribution $D_{g_{i}^{k}}\,$ is fitted based on the n expressions of $g_{i}^{k}$in the reference samples {*S*_1_,*S*_2_,⋯,*S*_*n*_} (Figure 1B).

[Step 4]. Construct a vector *P* of each local network *N*^*k*^(*k* = 1,2,⋯*Q*) for a single sample *S*_case_ of an individual. For the local network *N*^*k*^ (the local network centered on *g*^*k*^), a vector $P=\left[\mathrm{state}\,\left(g^{k}\right),\mathrm{state}\,\left(g_{1}^{k}\right),\cdots ,\mathrm{state}\,\left(g_{m}^{k}\right)\right]$ is obtained, in which each element is 0 or 1, that is, if the expression $g_{i}^{k}$ of *S*_case_ falls into the small probability interval of Gaussian distribution $D_{g_{i}^{k}}$, $\mathrm{state}\,\left(g_{i}^{k}\right)$ is 1, otherwise it is 0. Then the vector *P* of each local network*N*^*k*^ can be defined as follows:

(1) $$P=\left[\begin{array}{c} \begin{array}{c} p_{0}\\ p_{1}\\ \vdots \end{array}\\ p_{m} \end{array}\right]$$

With:

$$p_{0}=\frac{\mathrm{state}\,\left(g^{k}\right)}{\mathrm{state}\,\left(g^{k}\right)+\sum_{j=1}^{m}\mathrm{state}\,\left(g_{j}^{k}\right)},$$

$$p_{i}=\frac{\mathrm{state}\,\left(g_{i}^{k}\right)}{\mathrm{state}\,\left(g^{k}\right)+\sum_{j=1}^{m}\mathrm{state}\,\left(g_{j}^{k}\right)},i=1,2,\ldots ,m$$

[Step 5]. Calculate local single-sample node entropy (local SNE) for each local network *N*^*k*^(*k* = 1,2,…*Q*). For the local network *N*^*k*^ (the local network centered on *g*^*k*^), based on Eq. (1), the local SNE of it can be obtained as follows:

(2) $$H^{n}\,\left(g^{k}\right)=-\frac{1}{\left(m+1\right)}\,\sum_{i=0}^{m}p_{i}\,\log\left(p_{i}\right)$$

where constant *m* is the number of first-order neighbor of *g*^*k*^.

[Step 6]. Calculate the sum of *H*^*n*^(*g*^*k*^) for the top 5% local networks with the largest local SNE scores, i.e.,

(3) $$H\,\left(t\right)=\sum_{k=1}^{L}H^{n}\,\left(g^{k}\right)$$

where constant **L** is the number of the top 5% local networks. In Eq. (3), *H*(*t*)quantifies the overall perturbation triggered by a single case sample.

According to the DNB theory, when the system approaches the critical state, the nodes which falls into the small probability interval of background distribution will cause the increase of local SNE score *H*^*n*^(*g*^*k*^) in Eq. (2). Thus, a sharp peak in the SNE curve will show up when the early warning signals arrive.

### Data Processing and Functional Analysis

The SNE method has been applied to four unrelated real datasets from TCGA database^1^. These datasets included both cancer and cancer-adjacent samples. The cancer samples were divided into different stages according to the clinical information from TCGA and the samples lacking vital information are ignored. The cancer samples in ESCA dataset were grouped into six stages (stage I, IIA, IIB, IIIA, IIIB, and IV). The cancer samples of UCEC were classified into eight stages (stage IA, IB, IC, IIA, IIB, IIIA, IIIB, and IV). For READ and HNSC, the cancer samples were grouped into four stages, i.e., stage I, stage II, stage III, stage IV. The cancer-adjacent samples were viewed as reference samples.

The molecular global template network is built by the following steps:

(i) The biomolecular protein-protein interaction (PPI) network for *Homo sapiens* was downloaded from the functional protein association networks^2^ (Szklarczyk et al., 2015). All interaction used for discussion was picked with a high confidence (higher than 0.7). The transcriptional regulations were retrieved from transcriptional regulatory element database (rulai.cshl.edu/cgi-bin/TRED/tred.cgi?process=home). In this work, the linkage information together without redundancy was integrated into a whole molecular interaction network with 65,625 functional edges and 11,451 molecules.
(ii) All genes were mapped to the integrated network to extract the related linkages.
(iii) The molecular global template network was constructed based on all related linkages.

All pathways information come from Kyoto Encyclopedia of Genes and Genomes (KEGG^3^) and Reactome Pathway Analyzer (Fabregat et al., 2017).

The functional results was obtained using web service tools from the Gene Ontology Consortium (GOC^4^) and client software Ingenuity Pathway Analysis (IPA^5^). The enrichment analysis was based on Metascape (Zhou et al., 2019) and ClusterProfiler package (Yu et al., 2012) and some of gene information was extract from Human Cancer Metastasis Database (HCMDB^6^) and Precision Medicine Knowledge Base (Yu et al., 2019).

The differential expression and cox-survival analysis was performed in R statistical environment^7^ with DESeq2 package (version 1.26.0) (Love et al., 2014) and Survival package (version 3.1-11)^8^. Statistically significant results (*p* < 0.05) were included from both of them in the discussion of the article.

## Results

### Classic Gene Regulatory Network Model Shows the Utility of the SNE Algorithm

**FIGURE 2:**
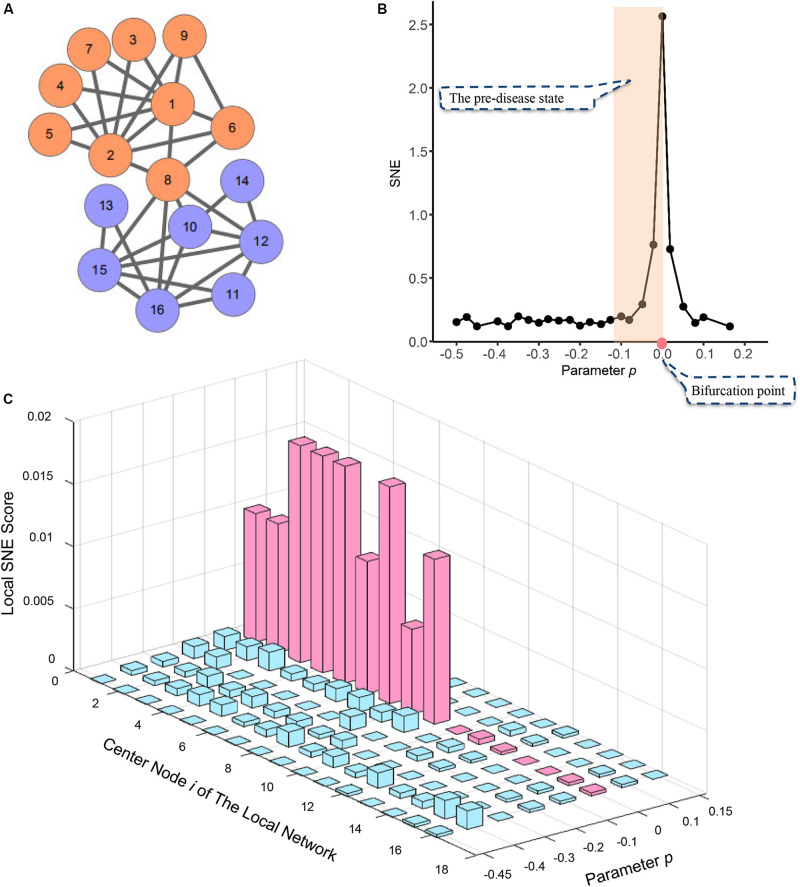
The application of SNE method in numerical simulation. **(A)** The numerical simulation is based on a model of a 16-node regulatory network. **(B)** The curve of SNE score H defined in Eq. (3) suddenly increases when the system is near the critical point (*p* = 0), which is viewed as a state transition at a bifurcation point. **(C)** The graph shows the dynamical changes of local SNE score defined as in Eq. (2) respectively, for 16 local networks. It can be seen that those local networks centered in the DNB members drastically increase when the system is near the critical point at *p* = 0, which provides the early warning signals of the imminent critical transition.

To validate the proposed SNE algorithm, a simple, simulated model network contains sixteen nodes (Figure 2A) was employed. In order to make the simulation model into a complete time series, a numerical simulation dataset was generated from the model network with varying parameter *p* ranging from −0.5 to 0.2.

A sudden increases of the single-sample node entropy (SNE) represented the imminent critical point when the system was near the parameter value *p* = 0 (Figure 2B). This suggests the system approached the pre-set tipping point. However, when the system was not critical, all the local SNE scores are at a low level (Figure 2C). Thus, numerical simulation model illustrates our SNE method can accurately detect the early warning signal when the system is about to go critical, even in such a tiny network.

### Identification of Critical Stage During Cancer Progression by SNE Methods Using TCGA Datasets

To validate the effectiveness of SNE method in identifying the pre-deterioration stage in cancer, we applied our model on distinct cancer cohort datasets. Using the single cancer sample, the local SNE score defined in Eq. (2) for each local network was calculated and ranked. Top 5% highest-ranked local SNE scores were chosen as the global SNE score.

**FIGURE 3:**
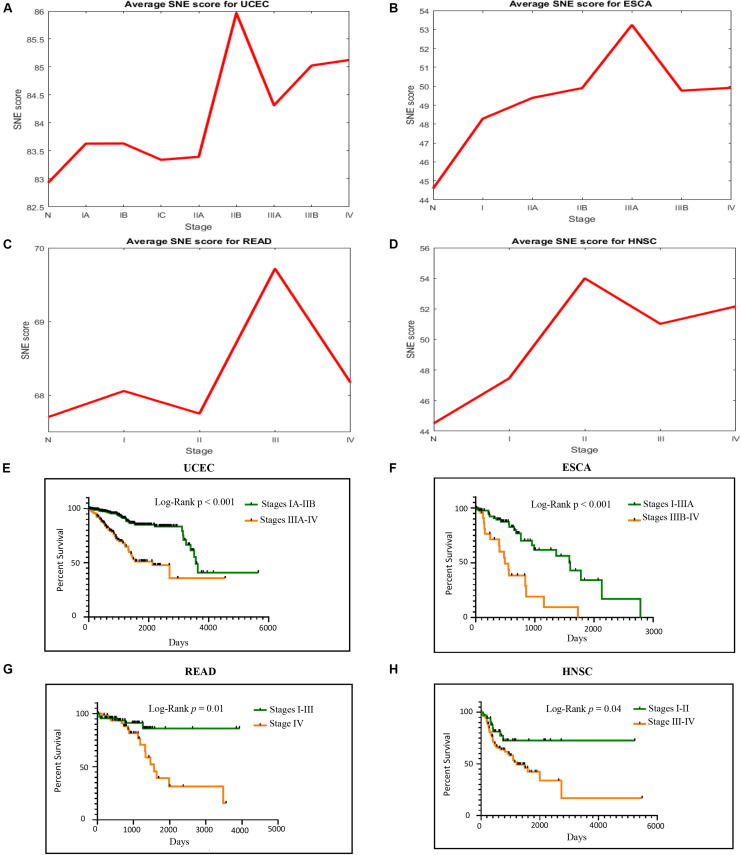
Cancer progression causes both sudden strike of SNE score and significant bad prognosis. **(A–D)** SNE score raises when meeting the clinical critical time in four TCGA cancer datasets. **(E–H)** Patients’ goes obviously spectated way whether they go through the critical point or not.

**FIGURE 4:**
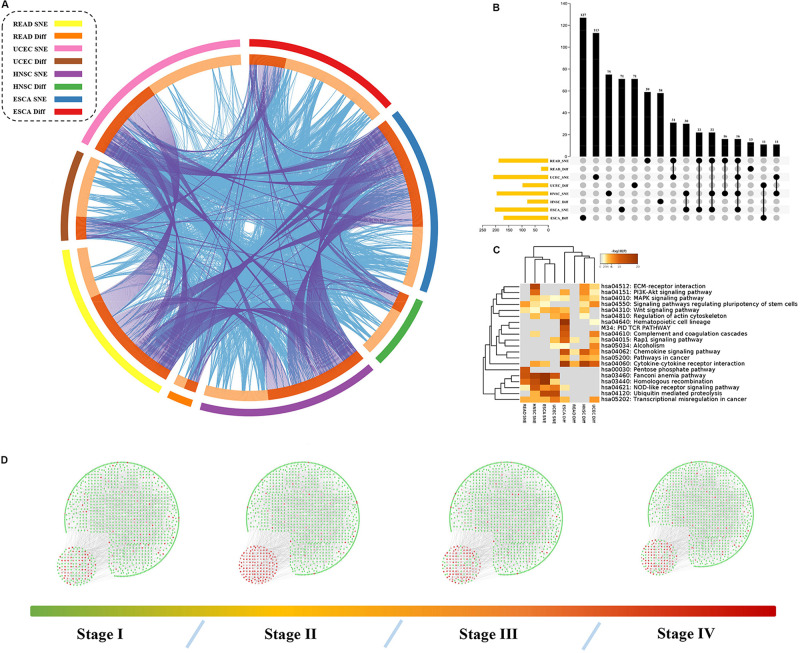
Hub genes, together with DEGs, are involved in important biological processes in cancer. **(A)** The identity of network node genes and DEG only overlap a little but biological function does not, the outer ring represent different group of genes and the inner ring represent their identity and function. The identities link with each other with purple lines and functions are in blue. **(B)** Exact number of gene overlap across all groups. **(C)** Function analysis shows that different gene set may play a different role in cancer progress. **(D)** Overall PPI network changes sharply in tipping point in HNSC.

Our algorithm got similar results in TCGA data as in simulated data, the critical time point for ECSA was identified in stage IIIA, that of UCEC was in stage IIB, that of READ was in stage III and HNSC was in stage II (Figures 3A–D). The PPI network also had a significant spatial-temporal change pattern and became chaotic during the critical stage (Figure 4D), which provided a dynamic change of molecular network from a global perspective. The critical stage in real data was also a valuable indicator for patient’s survival (Figures 3E–H). Therefore, these early warning signals detected during the cancer progression might contributed to clinical use.

### Dynamic Changes of Node Entropy Reveal Hidden Genes and Prognosis Biomarkers in Cancer Signal Regulation

To further explore “dark matter” in regulation factors of cancer related pathways, we compared these node genes with differential expressed genes in these datasets (see Supplementary Table S1). There were only a few intersections between network node genes and our DEGs, but there was closely relationship of their functions (Figures 4A,B).

**FIGURE 5:**
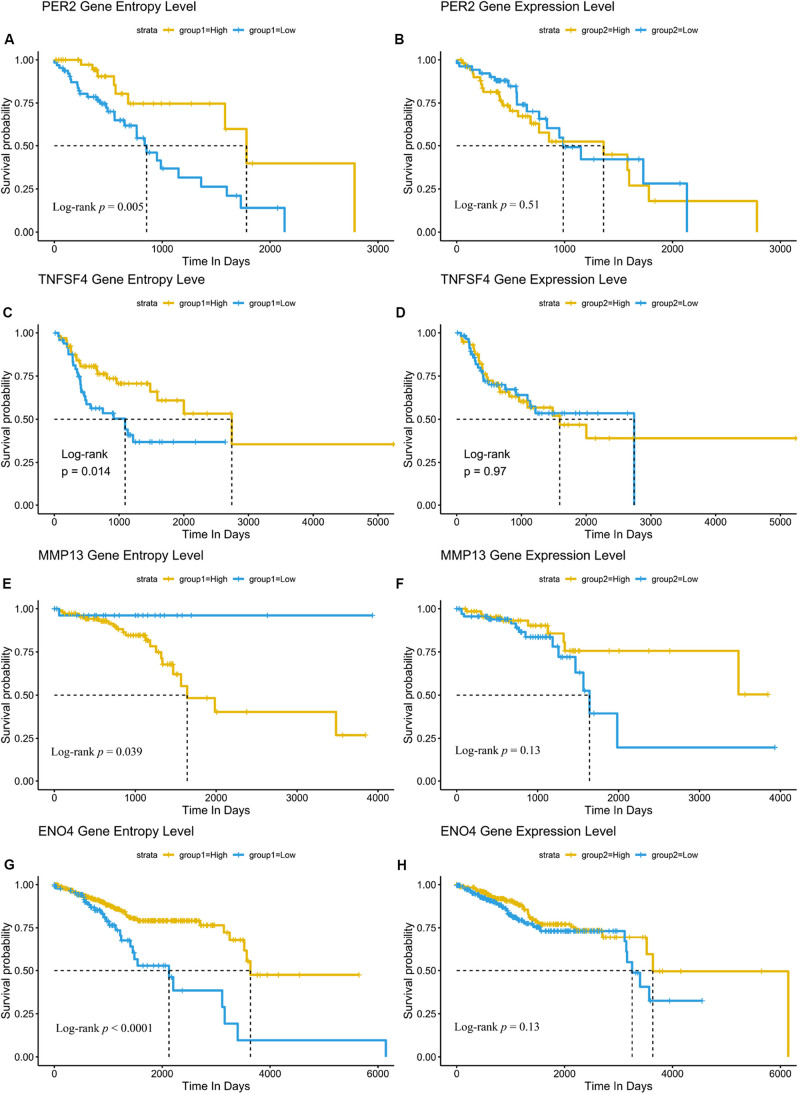
Network SNE is a supplementary prognosis signature for some novel genes than their expression. **(A–H)** Node entropy of SNE core genes *PER2* **(A,B)**, *TNFSF4* **(C,D)**, *MMP13* **(E,F)**, and *ENO4* **(G,H)** can be complementary markers of cancer prognosis compared with their expression level. Other markers with their significance are included in Supplementary Table S2.

Our results also showed that these genes were enriched in PI3K/Akt pathway, Wnt pathway or other pathways related to cancer (Figure 4C). Here, we focused on the role of the SNE gene in cancer treatment or progression. For example, *UBE2B* has been reported to be related to the resistance of platinum-based drugs (Sanders et al., 2017). Expression of the clock gene *PER2* is reported to play a key role in the occurrence and development of HNSCC and has not been associated with patient prognosis (Wang et al., 2017). Yet we also found *PER2* played an important role in the progression of ESCA and was not associated with patient prognosis. Expression of *TNFSF4* can affect the function of T cell activation through its specific receptor *TNFRSF4* (Graham et al., 2008), and is thought to contribute to T cell-dependent immunotherapy (Fu et al., 2020). The *MMP13* is vital in preparing for cancer metastasis (Liu and Cao, 2016). Importantly, further analysis showed *PER2*, *TNFSF4*, *MMP13* and other 52 genes were all strong related to patients’ survival based on signal entropy but not expression levels (Figures 5A–H and Supplementary Table S2). Therefore, we found that the signal entropy of some particular genes constructed in our algorithm could be indicator of genetic importance and supplement for patients’ prognosis.

### Hub Genes Locate Upstream in Cancer-Related Signaling Pathways and Trigger Downstream Differential Genes

To further clarify the association between these core genes fluctuated in critical stages and the differential expressed gene, we focused on several pathways most related to cancer progression and treatment (Perry et al., 2020; Yu et al., 2020). Compared with DEGs, SNE core genes occupied upstream or core positions in many pathways involving in cancer cell infiltration, proliferation or treatment (see Supplementary Figures S1–S4). In addition, we found the SNE core genes controlled the gating position of almost all upstream signal inputs into the Wnt pathway in HNSC, showing a completely control of Wnt signal initiation. Yet in READ, the SNE core genes only processed part of the input signals through canonical pathway, while the inputs through PCP pathway and Wnt/Ca 2^+^ pathway were almost unrestricted. In ESCA, we found that almost all genes controlling signal inputs were DEGs, meaning that Wnt signaling pathway might be out of control after critical stage. These differences in signal control could partially explain the cancer heterogeneity in molecular level. At the same time, we found *DAAM1* and *NKD2* gene, two of our hub genes involved in Wnt pathway, worked as intermediate elements in signal regulation across different cancer. Overexpression of *DAAM1* is thought to be related to breast cancer metastasis and bad prognosis (Mei et al., 2020). In addition, silencing *NKD2* can promote the progression of esophageal cancer by activating Wnt pathway in, which is similar to our findings (Cao et al., 2016).

In summary, the hub genes act as gatekeepers or key conducting elements in regulating cancer related signaling pathways. A sudden change of them at a specific time could be the reason for the emergence of DEGs, implying some valuable new targets for some specific pathways in drug development.

### Switch of Non-specific Gene Network in DNA Damage Repairing Pathway Drives Cancer Progression

**FIGURE 6:**
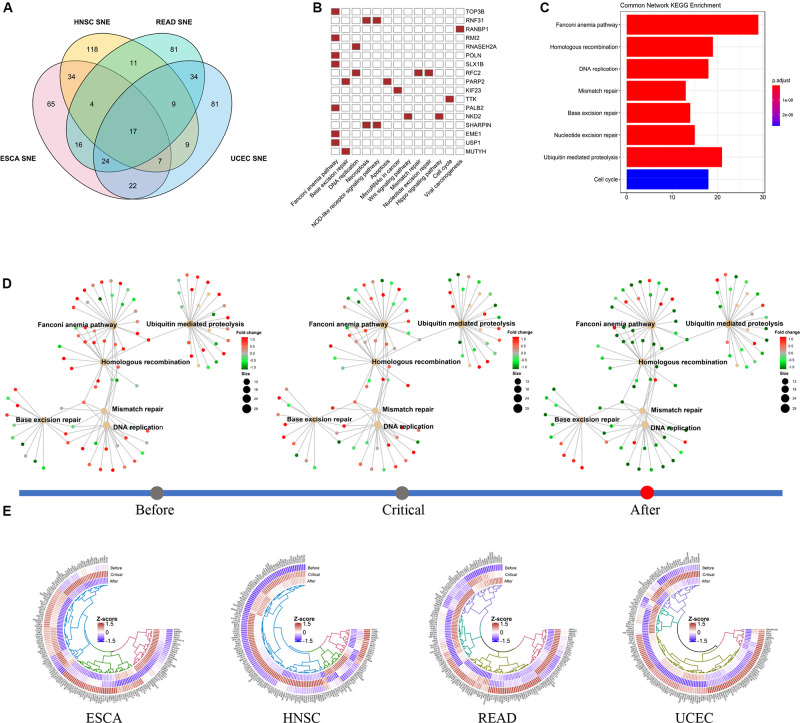
Functional switch of common SNE gene network in different cancer cohort. **(A)** In total, we identified 17 non-specific cancer SNE core genes. **(B)** Non-specific core genes are directly involved in multiple cancer-related pathways, particularly multiple signaling pathways associated with DNA repair. **(C)** The non-specific core genes and their 1st ranking gene interactive network are also enriched in multiple pathways related to DNA repair. **(D)** In the TCGA-ESCA cohort data, multiple DNA repair functions were turnover before and after the tipping point. **(E)** The expression pattern of the gene interactions network was significantly reversed with heterogeneity across multiple cancer between before- and after- critical stage. In addition, some of the genes do not significant changes in expression level in pre-deterioration stage.

To reveal the factors that might promote cancer progression across cancer types, we studied the SNE genes in four cancer in common and their first ranking interacted gene network (see Supplementary Table S3). We found that most of the common genes and the most relevant gene networks are involved in the DNA damage and repair process (Figures 6A–C). At the same time, we found the expression patterns of these networks involved in different DNA-damage-repair processes clearly switched before and after critical stage. We found some pathways, such as Fanconi anemia pathway, Base excision repair pathway, change from active to closed in TCGA-ESCA cohort, while some other pathway, such as DNA replication pathway, acted opposite in TCGA-READ and TCGA-UCEC cohort (Figure 6D).

We found that the expression pattern of the gene network was generally altered before and after the critical point, while the expression of some genes was not significantly different. Besides, we noted that there was inter-cancer heterogeneity in the conversion of network expression patterns. Same pathways there were though, the changing pattern for them were not identical in four cancer (Figure 6E).

## Discussion

RNA-seq has become a basic technology for cancer research, which provides important information for the development of a large number of targeted drugs (Stark et al., 2019). Since entropy has always been the foundation of informatics, projecting informatics theory to study how cells process and transmit information from environment has become important in cancer research (Banerji et al., 2015; Zielińska and Katanaev, 2019).

In our research, we have developed an algorithm named single-sample node entropy using only a single sample to measure cellular signaling activity in cancer critical stages. It is a model-free method that does not require any model training process. We have confirmed the stability and sensitivity in the analysis of simulation data and TCGA cohort data in our study. Using this model, we identified the tipping point of cancer progression, and found their importance to patient survival as expected. We also found some hub genes were highly connected to some important biological functions, such as cancer cell proliferation and invasion. Compared with differential expression analysis, our hub genes mostly acted as gatekeepers or core relays in multiple important pathways, such as *FZD8/9*, controlling the entry of canonical Wnt pathway, or *RRAS2*, which dominate Ras signaling pathway and proven to be associated with anti-breast cancer drug tamoxifen (Mendes-Pereira et al., 2012). Thus, by appropriately adjust the expression level of these hub genes, we may easily affect their downstream genes, for example, MYC or MAPK gene family in some important signaling pathways to further influence cancer growth. We noticed some genes’ node entropy was significantly related to patient’s survival, but their expression was not, suggesting we may obtain additional prognostic biomarker using our model. Therefore, if we obtained sufficient clinical patient samples, we had accurately measured the ability of node entropy as prognostic factor. Finally, we identified a series of SNE core genes associated with DNA repair, in which the *MUTYH* gene and the *PARP2* gene have been shown to be closely related to two approved anti-cancer drug mechanisms, Cisplatin and Olaparib (Khrunin et al., 2014; Verhagen et al., 2015), suggesting that these non-specific genes also have the potential to become drug targets.

However, our method also has some limitations. Our algorithm, based on complete time series data, is not suitable for sporadic disease. Moreover, although our method is based on a single sample in theory, sometimes it is still a challenge in collecting high-quality clinical samples to avoid accidental errors. So, the quality of clinical samples may affect the stability of the results. Besides, most hub genes have a small difference in expression level. Thus, it is difficult to confirm our results in experiments. To fully validate the function of hub genes, experiments in protein level may be required. In addition, although we found that the imbalance of the DNA damage and repair may be the cause of cancer progression, the complex interaction between those pathways is still unclear. More importantly, this is not the only factor that may promote cancer progression. Many other reasons have also been studied, such as metabolic disorder or cancer micro environment (Li et al., 2019; Wang et al., 2019). Finally, the TCGA data lacks more detailed clinical data, such as a more detailed division or an updated version of the cancer stages, which makes our results applicable only in a relatively rough range.

Despite all these, we should also notice our single-sample model may be useful to study other diseases, such as neurodegenerative diseases. It is very difficult to obtain enough samples for these slowly progressive disease. In addition, entropy has already become an important indicator of cell development potential and single cell cluster purity (Fabregat et al., 2017; Siegel et al., 2019), indicating that our method based on node entropy may also be important in single-cell analysis, especially in embryonic development or dynamic changes of immune cells (Chen et al., 2018; Hedlund and Deng, 2018).

## Conclusion

In this study, we developed a method for mining key point of cancer entities progression based on single-sample node entropy. This method can be applied robustly both in simulated regulatory network data and transferred interaction network data form cancer cohort entities.

Our algorithm has been verified in four independent TCGA data sets. We clarify the significance of these vital points for patients’ survival. In addition, the core network gene set could be a supplement for traditional statistical-based difference analysis to a certain extent. The results are also strongly linked to pathways important for cancer proliferation, invasion and metastasis, providing a “cause” perspective to cancer advancing. Finally, we focus on the potential value of gene network model switching across cancer types for cancer development and therapy and provide some potential drug targets.

All in all, identification of entropy hub genes in critical stages could give us new insights in drug development and clinical intervention.

## Data Availability Statement

All data can be obtained here (*https://github.com/BIOBRICK/Project-alpha*), and it also contains the script used to complete the full analysis process. Using the script in your own research does not require the author’s consent.

## Author Contributions

FL and RL conceived and designed the study. CH and JZ were involved in computational analysis. All authors wrote this manuscript and read and approved the manuscript.

## Conflict of Interest

The authors declare that the research was conducted in the absence of any commercial or financial relationships that could be construed as a potential conflict of interest.

## Footnotes

1 http://cancergenome.nih.gov

2 https://string-db.org

3 https://www.kegg.jp/

4 http://geneontology.org

5 http://www.ingenuity.com/products/ipa

6 http://hcmdb.i-sanger.com

7 https://www.r-project.org/

8 https://CRAN.R-project.org/package=survival
