# Supplementary figures and images for: Single-Sample Node Entropy for Molecular Transition in Pre-deterioration Stage of Cancer
Source: Front Bioeng Biotechnol. 2020 Jul 14;8:809. doi: 10.3389/fbioe.2020.00809 (PMC7381145; doi:10.3389/fbioe.2020.00809)

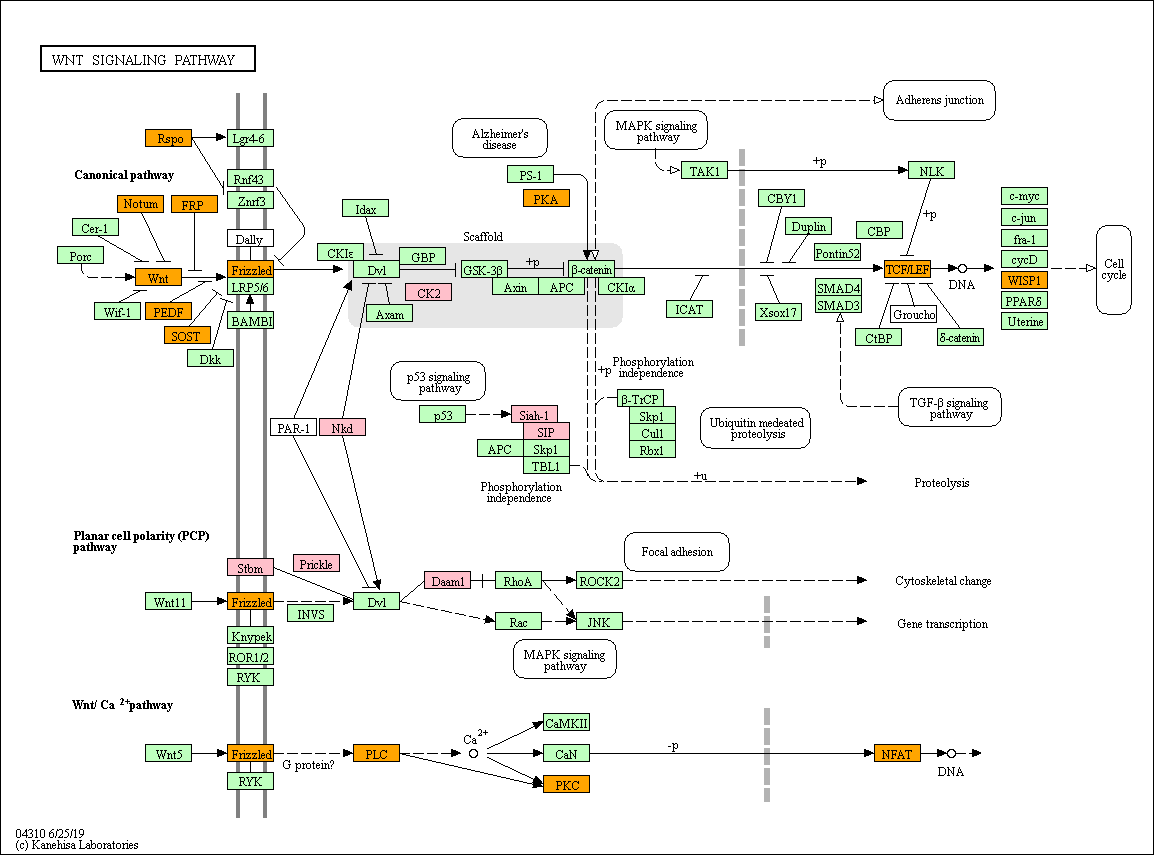

Supplement: Supplementary file 1 [file Image_1.PNG]

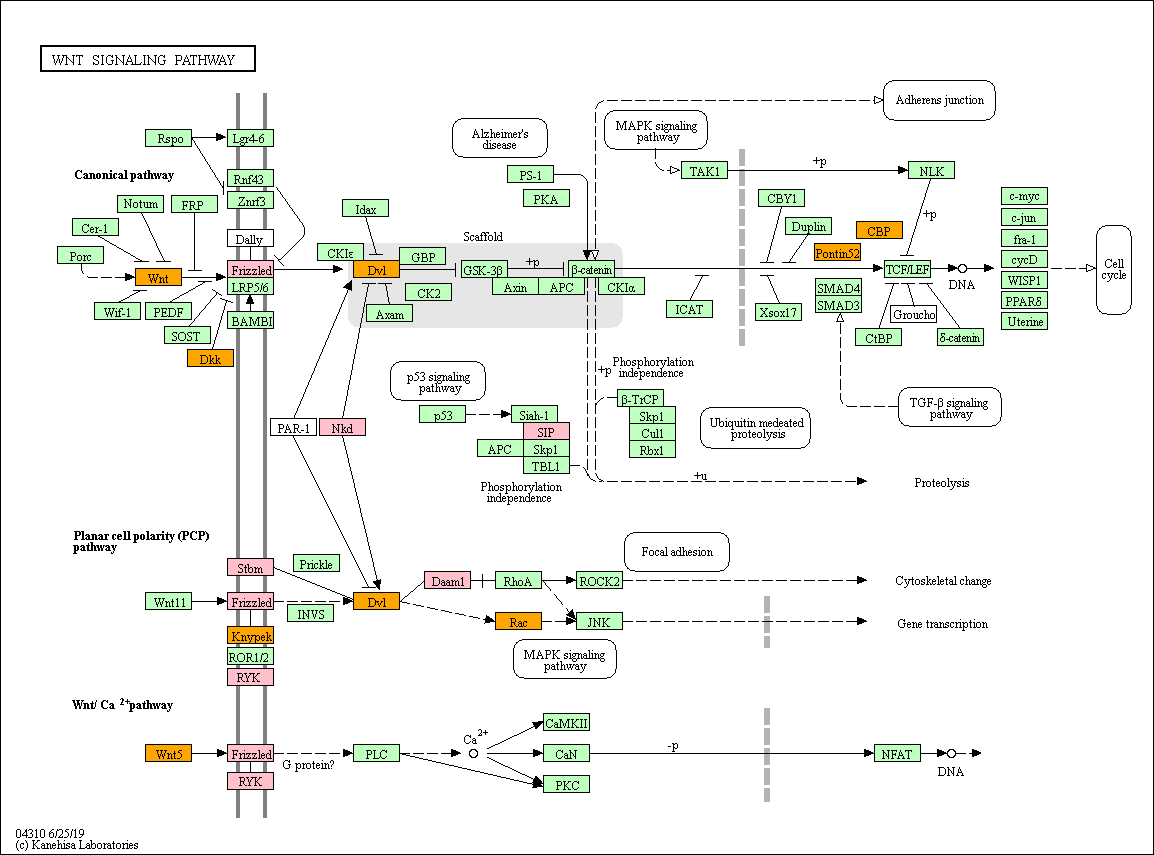

Supplement: Supplementary file 2 [file Image_2.PNG]

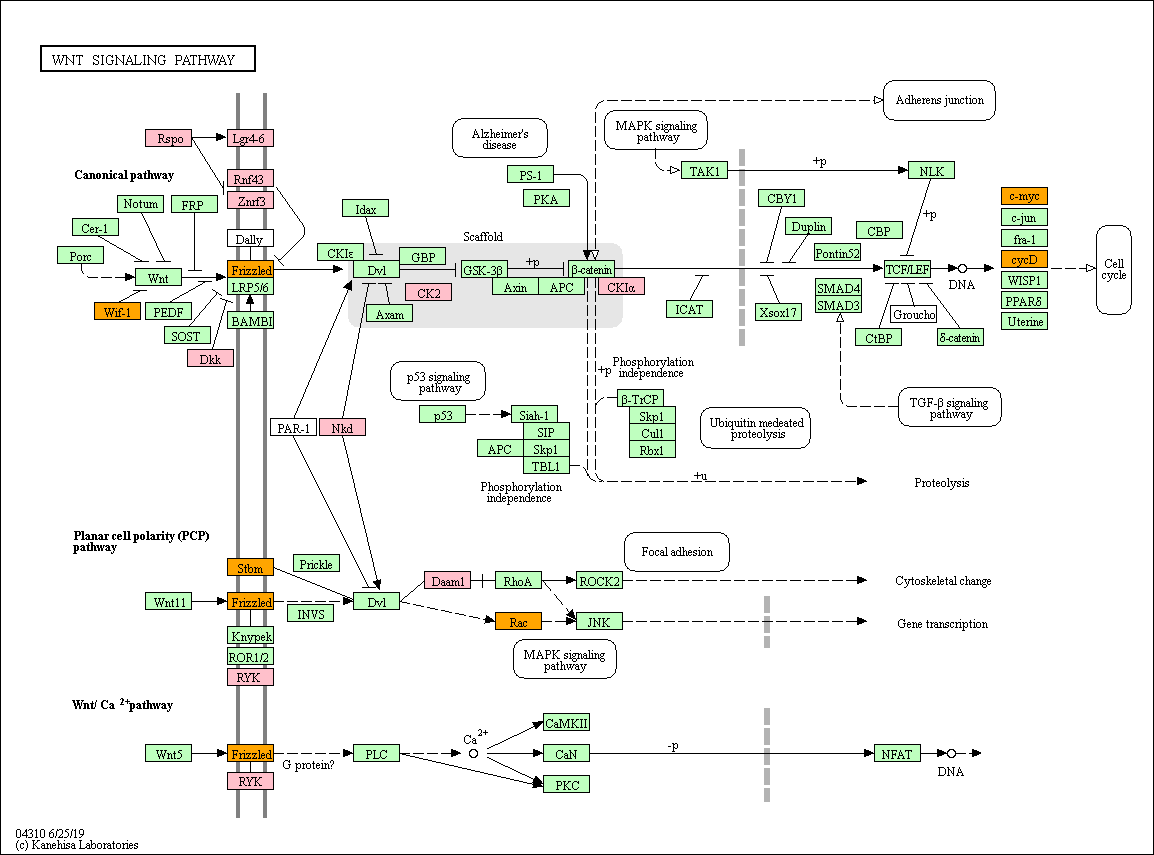

Supplement: Supplementary file 3 [file Image_3.PNG]

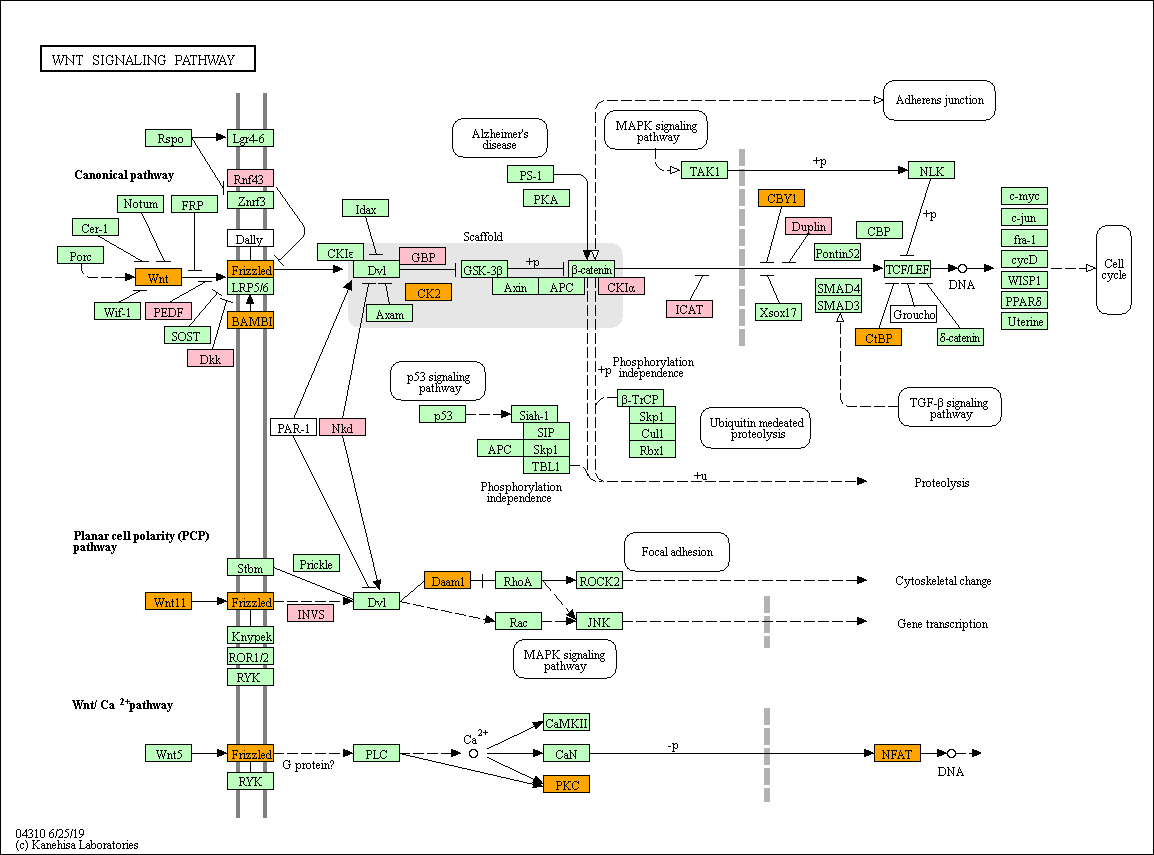

Supplement: Supplementary file 4 [file Image_4.PNG]
