# Supplementary material for: Single-Sample Node Entropy for Molecular Transition in Pre-deterioration Stage of Cancer
Source: Front Bioeng Biotechnol. 2020 Jul 14;8:809. doi: 10.3389/fbioe.2020.00809 (PMC7381145; doi:10.3389/fbioe.2020.00809)
Supplement: Supplementary file 8 [file Presentation_1.pdf]

Supplementary Material

1 Supplementary Data

Suppl This manuscript does not contain any additional data, all raw data was obtained from the GDC TCGA Data Portal, and intermediate data can be obtained entirely from the script provided in the manuscript.

2 Supplementary Figures and Tables

2.1 Supplementary Figures

Supplementary Figure 1. In the TCGA-ESCA cohort, the SNE core genes correspond to the signaling positions of DEGs in the Wnt pathway, with the SNE core genes identified by pink and DEGs identified by orange. The map was generated by the SNE core genes with a list of DEGs in KEGG Mapper.

Supplementary Figure 2. In the TCGA-HNSC cohort, the SNE core genes correspond to the signaling positions of DEGs in the Wnt pathway, with the SNE core genes identified by pink and DEGs identified by orange. The map was generated by the SNE core genes with a list of DEGs in KEGG Mapper.

Supplementary Figure 3. In the TCGA-READ cohort, the SNE core genes correspond to the signaling positions of DEGs in the Wnt pathway, with the SNE core genes identified by pink and DEGs identified by orange. The map was generated by the SNE core genes with a list of DEGs in KEGG Mapper.

Supplementary Figure 4. In the TCGA-UCEC cohort, the SNE core genes correspond to the signaling positions of DEGs in the Wnt pathway, with the SNE core genes identified by pink and DEGs identified by orange. The map was generated by the SNE core genes with a list of DEGs in KEGG Mapper.

2.2 Supplementary Table

Supplementary Table 1. A list of core genes generated by SNE and a list of differential genes generated by differential analysis.

Supplementary Table 2. List of genes significantly associated with patient prognosis and *p*-value.

Supplementary Table 3. Common SNE core genes and their first ranking interacted networks genes.
